# Supplementary material for: Glycaemic Imbalances in Seizures and Epilepsy of Paediatric Age: A Literature Review
Source: J Clin Med. 2023 Mar 29;12(7):2580. doi: 10.3390/jcm12072580 (PMC10095009; doi:10.3390/jcm12072580)
Supplement: Supplementary file 1 [file jcm-12-02580-s001.zip › jcm-2277544-supplementary.pdf]

Supplementary material

1. A practical clinical definition of epilepsy

Epilepsy was defined conceptually in 2005 as a disorder of the brain characterized by an enduring predisposition to generate epileptic seizures. This definition is usually practically applied as having two unprovoked seizures>24 h apart. The International League Against Epilepsy (ILAE) accepted recommendations of a task force altering the practical definition for special circumstances that do not meet the two unprovoked seizures criteria. The task force proposed that epilepsy be considered to be a disease of the brain defined by any of the following conditions: (1) At least two unprovoked (o rreflex) seizures occurring>24 h apart; (2) one unprovoked (or reflex) seizure and aprobability of further seizures similar to the general recurrence risk (at least 60%) after two unprovoked seizures, occurring over the next 10 years; (3) diagnosis of an epilepsy syndrome.

Modified from: [164].

2. Stages of childhood development

- New-born infant or neonate: child under 28 days of age
- Infant: 0-1 year of age
- Toddler child: 1-2 years of age
- Pre-schooler child: 2-6 years of age
- School-aged child: 6-12 years of age
- Adolescent: 12-18 years of age

Modified from [165].

3. Criteria for the diagnosis of diabetes

Diabetes may be diagnosed based on plasma glucose criteria, either the fasting plasma glucose (FPG) value or the 2-h plasma glucose (2-h PG) value during a 75-g oral glucose tolerance test (OGTT), or A1C criteria

- FPG ≥126 mg/dL (7.0 mmol/L). Fasting is defined as no caloric intake for at least 8 h.*
  OR
- 2-h PG ≥200 mg/dL (11.1 mmol/L) during OGTT. The test should be performed as described by WHO, using a glucose load containing the equivalent of 75 g anhydrous glucose dissolved in water*
  OR
- A1C ≥6.5% (48 mmol/mol). The test should be performed in a laboratory using a method that is NGSP certified and standardized to the DCCT assay*
  OR
- In a patient with classic symptoms of hyperglycemia or hyperglycemic crisis, a random plasma glucose ≥200 mg/dL (11.1 mmol/L).

DCCT, Diabetes Control and Complications Trial; FPG, fasting plasma glucose; OGTT, oral glucose tolerance test; WHO, World Health Organization; 2-h PG, 2-h plasma glucose. *In the absence of unequivocal hyperglycemia, diagnosis requires two abnormal test results from the same sample or in two separate test samples.

Modified from [148].

4. Management of Neonates, Infants,and Children with a Persistent Hypoglycemia Disorder

4.1. For neonates with a suspected congenital hypoglycaemia disorder and older infants and children with a confirmed hypoglycemia disorder, we recommend that the goal of treatment be to maintain a PG concentration >70 mg/dL(3.9 mmol/L). GRADE 1++00.

4.2. For high-risk neonates without a suspected congenital hypoglycemia disorder, we suggest the goal of treatment beto maintain a PG concentration >50 mg/dL (>2.8 mmol/L)for those aged <48 hours and >60 mg/dL (>3.3 mmol/L)for those aged >48 hours. GRADE 2+000.

4.3. We recommend an individualized approach to management with treatment tailored to the specific disorder, tak-ing into account patient safety and family preferences. Ungraded best practice statement

Modified from [23].

Short-term treatment of hypoglycemia consists of an intravenous (IV) bolus of dextrose 10% 2.5 mL/kg. The critical sample should be drawn before the glucose is administered. After the bolus is administered, an IV infusion that matches normal hepatic glucose production (approximately 5-8 mg/kg/min in an infant and about 3-5 mg/kg/min in an older child) should be continued. This should be adjusted to maintain the plasma glucose level at more than 3 mmol/L. Children with hyperinsulinemia may have much higher needs. Glucagon infusion at rates of 0.005-0.02 mg/kg/h should be used as a temporary treatment in children with hyperinsulinism in whom adequate amounts of dextrose cannot be given. It can cause a rash and decreased appetite if used over the long term.

Modified from [166] and [167].
